# Fungal Communication Requires the MAK-2 Pathway Elements STE-20 and RAS-2, the NRC-1 Adapter STE-50 and the MAP Kinase Scaffold HAM-5

**DOI:** 10.1371/journal.pgen.1004762

**Published:** 2014-11-20

**Authors:** Anne Dettmann, Yvonne Heilig, Oliver Valerius, Sarah Ludwig, Stephan Seiler

**Affiliations:** 1 Institute for Biology II – Molecular Plant Physiology, Albert-Ludwigs University Freiburg, Freiburg, Germany; 2 Institute for Microbiology and Genetics, University of Goettingen, Goettingen, Germany; 3 Freiburg Institute for Advanced Studies (FRIAS), Albert-Ludwigs University Freiburg, Freiburg, Germany; Duke University Medical Center, United States of America

## Abstract

Intercellular communication is critical for the survival of unicellular organisms as well as for the development and function of multicellular tissues. Cell-to-cell signaling is also required to develop the interconnected mycelial network characteristic of filamentous fungi and is a prerequisite for symbiotic and pathogenic host colonization achieved by molds. Somatic cell–cell communication and subsequent cell fusion is governed by the MAK-2 mitogen activated protein kinase (MAPK) cascade in the filamentous ascomycete model *Neurospora crassa*, yet the composition and mode of regulation of the MAK-2 pathway are currently unclear. In order to identify additional components involved in MAK-2 signaling we performed affinity purification experiments coupled to mass spectrometry with strains expressing functional GFP-fusion proteins of the MAPK cascade. This approach identified STE-50 as a regulatory subunit of the Ste11p homolog NRC-1 and HAM-5 as cell-communication-specific scaffold protein of the MAPK cascade. Moreover, we defined a network of proteins consisting of two Ste20-related kinases, the small GTPase RAS-2 and the adenylate cyclase capping protein CAP-1 that function upstream of the MAK-2 pathway and whose signals converge on the NRC-1/STE-50 MAP3K complex and the HAM-5 scaffold. Finally, our data suggest an involvement of the striatin interacting phosphatase and kinase (STRIPAK) complex, the casein kinase 2 heterodimer, the phospholipid flippase modulators YPK-1 and NRC-2 and motor protein-dependent vesicle trafficking in the regulation of MAK-2 pathway activity and function. Taken together, these data will have significant implications for our mechanistic understanding of MAPK signaling and for homotypic cell–cell communication in fungi and higher eukaryotes.

## Author Summary

Appropriate cellular responses to external stimuli depend on the highly orchestrated activity of interconnected signaling cascades. One crucial level of control arises from the formation of discrete complexes through scaffold proteins that bind multiple components of a given pathway. Central for our understanding of these signaling platforms is the archetypical MAP kinase scaffold Ste5p, a protein that is restricted to budding yeast and close relatives. We identified HAM-5, a protein highly conserved in filamentous ascomycete fungi, as cell–cell communication-specific scaffold protein of the *Neurospora crassa* MAK-2 cascade (homologous to the budding yeast pheromone pathway). We also describe a network of upstream acting proteins, consisting of two Ste20-related kinases, the small G-protein RAS-2 and the adenylate cyclase capping protein CAP-1, whose signals converge on HAM-5. Our work has implications for the mechanistic understanding of MAP kinase scaffold proteins and their function during intercellular communication in eukaryotic microbes as well as higher eukaryotes.

## Introduction

Intercellular communication is critical for the survival of simple unicellular organisms such as bacteria and yeasts and is central for the development and function of multicellular plant and animal systems [1]–[4]. Cell-cell signaling and somatic cell fusion is also required to develop the interconnected mycelial network characteristic of filamentous fungi [5]. This feature is important for the fitness of the fungal colony by the shared use of information, nutrients and organelles between individual cells [6], [7]. Consequently, hyphal anastomosis is critical for host colonization and symbiotic interactions as well as for virulence of pathogenic species [8]–[12]. Hyphal fusion is comparable to homotypic cell fusion between genetically identical cells of higher eukaryotes, which results in the formation of multinucleate syncytia [13], [14]. Important examples for human biology are myoblast fusion during muscle differentiation, trophoblast fusion during placental development and osteoclast fusion during bone formation. Thus, fungal self-signaling may provide a powerful model for understanding molecular mechanisms of homotypic cell communication during animal and human tissue development.

In the ascomycete model mold *Neurospora crassa*, an unknown chemical ligand mediates chemotropic communication between genetically identical cells. Germinating spores mutually attract each other and subsequently fuse to generate an interconnected network of multinucleate cells that form the mycelial colony [5], [15]. This process of self-signaling is based on the oscillatory recruitment of the NRC-1–MEK-2–MAK-2 mitogen activated protein kinase (MAPK) cascade (homologous to the Ste11p-Ste7p-Fus3p mating pathway in budding yeast) and of SOFT, a protein of unknown molecular function, to the opposing tips of communicating germlings [16], [17]. The rapid alternation of these two different physiological states of “homing” cells likely reflects signal response and delivery, respectively. Although these findings resulted in a first qualitative model to describe the excitable behavior of the MAK-2 module [18], our understanding of oscillatory MAK-2 signaling is hampered by the fact that most components of the signaling machinery – including the postulated secreted signal and its cognate receptor(s), regulators of the MAPK cascade as well as most MAK-2 targets – are unknown.

*N. crassa* and other filamentous fungi possess G-protein coupled receptors, heterotrimeric G-proteins, STE20-related kinases, components of the cAMP machinery and Ras/Rho-type GTPase modules known to function upstream or in parallel of MAPK signaling in fungi and higher eukaryotes [19]–[22]. However, mutant analyses indicate that individual deletions of these components are dispensable for vegetative cell communication (summarized in [23]). We hypothesized that redundant functions between the mentioned proteins require additional approaches to classical mutant hunts in order to dissect MAK-2 signaling. In this study, we used a proteomics approach that allowed the identification of STE-50 as regulatory subunit of the Ste11p homolog NRC-1 and HAM-5 as cell-communication-specific scaffold protein of the MAPK cascade. Moreover we defined a network of proteins, consisting of two Ste20-related kinases, the small G-protein RAS-2 and the adenylate cyclase capping protein CAP-1, whose signals converge on the MAK-2 pathway.

## Results

### Composition of the MAK-2 cascade

**Figure 1:**
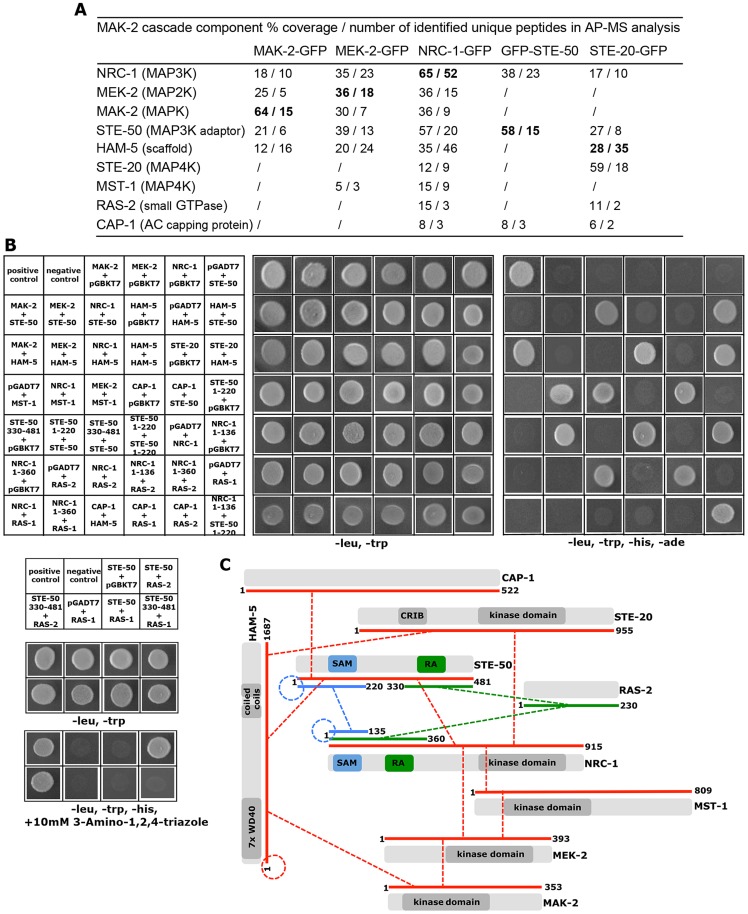
Interaction network of MAK-2 pathway components. (**A**) Proteins associated with the MAK-2 cascade were identified in affinity purification experiments coupled to mass spectrometry (AP-MS), and identified proteins were filtered against control purifications using GFP as bait (Table S1). Only proteins identified in two biological replicates and absent from the control data set are shown. Protein coverage by peptides and the number of identified total and unique peptides identified are given for the better of the two purifications. Bold numbers indicate the GFP-fusion protein used as bait. (**B**) Physical interactions between MAK-2 pathway components were mapped in yeast two-hybrid (Y2H) tests. The indicated constructs were co-expressed in strain AH109 and yeast growth was analyzed on the indicated selective media. The reciprocal Y2H assays are shown in Figure S2. (**C**) Summary schema of Y2H-based interactions of the indicated proteins and their domains. Color-coded lines below the protein schemas and dashed connectors indicate the used constructs and detected interactions, respectively.

A high-quality interactome of the MAK-2 MAPK module was generated by affinity purification experiments coupled to mass spectrometry (AP-MS) with strains expressing functional GFP-fusion proteins of the three kinases of the MAK-2 cascade (Table S1). Each of the three bait proteins recovered the tripartite kinase cascade with high stringency in the two biological replicate purifications performed, indicating the suitability of the approach. Moreover, we identified two proteins, STE-50 and HAM-5, which displayed tight interactions with all three kinases (Figure 1 A).

The remaining hits that associated with all three kinases were only identified with poor coverage and/or in one of the two purifications and thus could constitute contaminants. However, among them were PPG-1, PP2A-A, HAM-3 and MOB-3, components of the striatin-interacting phosphatase 2A and kinase (STRIPAK) complex that is required for fungal cell-cell signaling [24]–[29]. This suggests that at least some of the additional hits may represent dynamically, and thus weakly interacting components. Thus, we assayed available mutants defective for additional candidate proteins that associated with the three kinases for tropic interaction defects and determined that the casein kinase 2 heterodimer CKA/CKB-1, the serine/threonine kinase YPK-1, type V myosin/NCU01440, and the hypothetical protein NCU06265 (designated HAM-13) were required for proper cell communication. Consistent with these defects, we also detected reduced MAK-2 phosphorylation levels in cell extracts of these five mutant strains (Table 1; Figure S1). The combined proteomics data and mutant characteristics indicate a specific involvement of these proteins in cell-cell communication despite the fact that they were identified only with poor coverage in the AP-MS analysis. However, further experiments are required to determine their mechanistic mode of action and to exclude indirect effects of the mutations on MAK-2 pathway functionality. In addition, mutants defective for the hypothetical proteins NCU00627, NCU02606, NCU02972, and NCU08957 that also interacted with the entire kinase cascade displayed inconspicuous communication patterns and were thus not tested for MAK-2 phosphorylation, because they likely represented contaminants.

**Table 1:** Mutant characteristics of MAK-2 pathway components.

Strain (FGSC # or [reference])	Annotation	Cell communication 1 2	MAK-2 phosphorylation 2
wild type (#987)		95%±2	100%
ΔNCU00455 (#17041×wt)	MAP3K regulator STE-50	0%	0%
ΔNCU00627 (#16766)	hypothetical protein	96%±2	nd 3
ΔNCU00772 [47]	STE kinase MST-1	95%±3	99%±10
ΔNCU01440 (#11422)	type V myosin	9%±3 *	14%±8
ΔNCU01789 (#15045)	IDC1 related protein HAM-5	9%±1	18%±9
ΔNCU02606 (#17297)	hypothetical protein	95%±2	nd
ΔNCU02972 (#19746)	hypothetical protein	95%±3	nd
ΔNCU03124 (#17973 4)	casein kinase 2 alpha subunit CKA	12%±4	28%±6
ΔNCU05485 (#15567)	casein kinase 2 beta 1 subunit CKB-1	23%±2 *	22%±8
ΔNCU06265 (#11245)	hypothetical protein; HAM-13	33%±2	42%±5
ΔNCU07280 (#13416 4)	protein kinase YPK-1	14%±2 *	nd
ypk-1(16-19) [79]	protein kinase YPK-1	54%±8	20%±8
ΔNCU03616 (#12467)	Ras GTPase RAS-2/SMCO-7	11%±2	29%±10
smco-7 [49]	Ras GTPase RAS-2/SMCO-7	9%±3	37%±7
ΔNCU03894 (#11325)	STE kinase STE-20	77%±2	94%±5
Δste-20;Δmst-1 (this study)	STE kinase double mutant	69±5	93%±6
ΔNCU06500 (#16014 4)	Ras GDP-GTP exchange factor	14%±3	15%±9
cdc-25(7-10) ([79])	Ras GDP-GTP exchange factor	5%±2 *	nd
Δste-20;Δras-2 (this study)	ste-20 ras-2 double mutant	5%±3	21%±5
ΔNCU08008 (#12371)	adenylate cyclase capping protein CAP-1	28%±5	63%±8
ΔNCU08377 (#11514×wt)	adenylate cyclase CR-1	34%±5 *	37%±6
ΔNCU08957 (#19203)	hypothetical protein	97%±2	nd

1 assayed at 6 h post inoculation except for strains marked with *, which were analyzed 8 h post inoculation due to reduced germination rate and abnormal growth.

2 in %±SEM.

3 not determined.

4 heterokaryotic deletion strain.

Interestingly, we also determined that MAK-2, yet not the two upstream kinases interacted with multiple components of the nuclear import/export machinery (i.e. importin alpha, importin beta-1, importin beta-3, exportin-1, nuclear pore protein NCU01702; Table S1) and the two transcriptional regulators PP-1 and RCO-1, which were previously identified as key effectors of the MAK-2 pathway [30]–[32].

### STE-50 is required for NRC-1 activation

Budding yeast Ste50p functions as adaptor protein of the MAP3K Ste11p that connects heterotrimeric G-proteins and small GTPases with various MAPK cascades [33], [34]. This is achieved through the modular structure of Ste50p. The protein consists of an N-terminal protein interaction domain called the sterile alpha motif (SAM) [35], [36] and a C-terminal Ras association (RA) domain that can bind to small Ras and Rho-type GTPases and is required for membrane delivery of the Ste11p/Ste50p complex [37], [38]. Homologs in filamentous fungi are not as well characterized, but have been proposed to function as scaffold proteins in MAPK cascades [39]–[42]. Yeast two hybrid (Y2H) assays confirmed the physical interaction of STE-50 with itself and with NRC-1, yet not MEK-2 and MAK-2, through the SAM domains present in the two proteins (Figure 1 B, C; Figure S2). The presence of a stable STE-50/NRC-1 complex was further supported by the reciprocal AP-MS analysis of GFP-STE-50 interacting proteins, which revealed a stable interaction of STE-50 with NRC-1, but not other components of the MAPK cascade (Figure 1 A).

**Figure 2:**
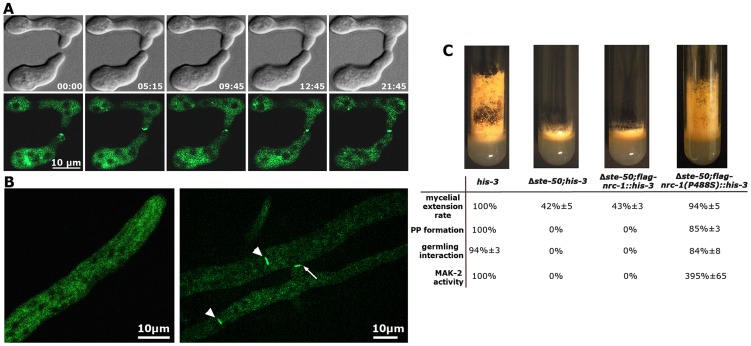
STE-50 functions as regulatory subunit of NRC-1. (**A**) GFP-STE-50 localizes in a dynamic manner to opposing tips of two communicating germlings and the site of cell-cell contact. The oscillation period is approximately three to five minutes. See Video S1 for time course. (**B**) GFP-STE-50 localizes in a diffuse cytosolic manner in non-communicating hyphae and is excluded from nuclei (left image). Moreover, GFP-STE-50 accumulates at septa (arrowheads) and the contact point (arrow) of communicating hyphal tips (right image). (**C**) Δ*ste-50* fully phenocopies Δ*mak-2* defects (see Figure S3 for comparative characterization of mutant defects), and the mutant characteristics are complemented by expression of a constitutive active NRC-1(P488S) allele in Δ*ste-50*. The slant images represent macroscopic appearance and conidiation pattern, while the Table summarizes rates of mycelial extension, protoperithecia (PP) formation, germling communication and MAK-2 activities of the indicated strains.

Strains expressing *gfp*-*ste-50* under its native promoter complemented the defects of the deletion strain, but barely showed any fluorescence, which was consistent with the weak expression profile of its binding partner NRC-1 [17]. Thus, we analyzed strains expressing the fusion construct under the control of the *Pccg-1* promoter. GFP-STE-50 displayed a subcellular dynamics similar to that previously described for the three kinases of the MAK-2 cascade [16], [17] and accumulated at the future fusion site after the two germlings had established physical contact (Figure 2 A). Consistent with the expression profile and localization pattern described for NRC-1 [17], we observed only weak cytoplasmic localization of GFP-STE-50 in mature hyphae and exclusion of the fusion protein from nuclei. However, GFP-STE-50 accumulated at septa and contact sites of fusing hyphae within an established colony (Figure 2 B).

We determined that a Δ*ste-50* deletion mutant fully phenocopied all defects described for defective MAK-2 signaling [31], [43], [44] and lacked detectable MAK-2 kinase activity (Table 1; Figure 2 C; Figure S3). Based on these characteristics of the mutant and the tight interaction of STE-50 with NRC-1, we hypothesized that STE-50 may function as regulatory subunit of the MAP3K. To test this, we expressed a recently generated [17], constitutive active NRC-1 version NRC-1(P488S) in Δ*ste-50*. This construct fully complemented all defects of the deletion strain and confirmed our hypothesis (Figure 2 B).

### HAM-5 functions as cell communication-specific scaffold of the MAK-2 module

*Ham-5* was originally identified in a genetic screen for *N. crassa* mutants that fail to undergo cell fusion [30]. Its closest homolog, *Podospora anserina* IDC1, is required for NADPH-oxidase-dependent nuclear accumulation of the cell wall integrity MAPK Mpk1 [45], but the mechanistic basis underlying this observation remains unresolved. MAK-2 activity was reduced to ca. 1/10 of wild type in the Δ*ham-5* mutant (Table 1; Figure S3), indicating significantly compromised, but not abolished MAK-2 pathway function in this strain. We observed increased mycelial extension rates of Δ*ham-5* when compared to Δ*ste-50* or Δ*mak-2*, residual tropic interactions (≤10% of wild type) and the retained capacity to produce low amounts of infertile protoperithecia (ca. 20% of wild type; Figure S3). Co-immunoprecipitation experiments were used to confirm the interaction of HAM-5 with all three kinases of the MAK-2 pathway Figure S2). In order to further dissect the physical interaction pattern of these proteins, we performed Y2H tests. In these assays, we determined that HAM-5, which contained seven N-terminally located WD40 repeats and two short coiled coil regions in the C-terminal region, was able to homo-dimerize and also interacted with STE-50 and with MAK-2, yet not with MEK-2 and NRC-1 (Figure 1 B, C; Figure S2).

**Figure 3:**
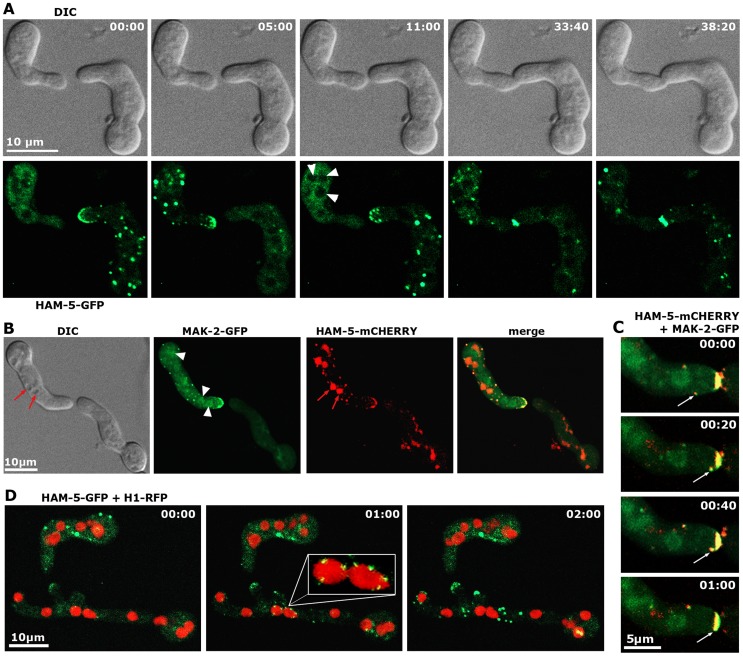
HAM-5 and MAK-2 dynamically co-localize in communicating germlings. (**A**) Motile HAM-5-GFP puncta appear in a dynamic manner at opposing tips and in the cell body of two communicating germlings with an oscillation period of approximately three to five minutes and subsequently accumulate at the contact point of the two tips. See Video S2 for time course. Note that the diffuse cytoplasmic label of HAM-5-GFP decreased during the formation of HAM-5 puncta. Arrowheads indicate nuclei, from which HAM-5 is excluded. (**B**) HAM-5 and MAK-2 co-localize to puncta at the cell tip and within the germling (marked by arrows). MAK-2, but not HAM-5 also accumulates in nuclei (marked by arrowheads). Note that mCHERRY-tagged HAM-5 is strongly targeted to vacuoles for degradation (marked by red arrows). See Video S3 for time course. (**C**) Movement of a HAM-5/MAK-2 double-labeled spot towards the cell tip. See Video S3 for time course. (**D**) HAM-5 puncta can associate with nuclear envelopes (enlarged insert). See Video S4 for time course. (**C**) Movement of a HAM-5/MAK-2 double-labeled spot towards the cell tip. See Video S3 for time course.

Functional *Pccg-1*-driven HAM-5-GFP displayed the predicted dynamic subcellular localization during germling communication and strictly co-localized with MAK-2 as dynamically forming intracellular complexes that associated with the communicating tips of the two cells with an oscillation period of three to five minutes (Figure 3 A, B). At least some of the tip-associated signal was generated by recruitment of cytosolic HAM-5- and MAK-2-containing puncta to the apex (Figure 3 C). Diffuse HAM-5-GFP label was excluded from nuclei, and bright HAM-5 puncta were not obviously associated with nuclei. However, we detected weakly labeled HAM-5 puncta that decorated nuclear envelopes (preferentially marking those nuclei that localized closely to the cell tip) simultaneously with the first appearance of tip-associated HAM-5 signal. (Figure 3 D).

**Figure 4:**
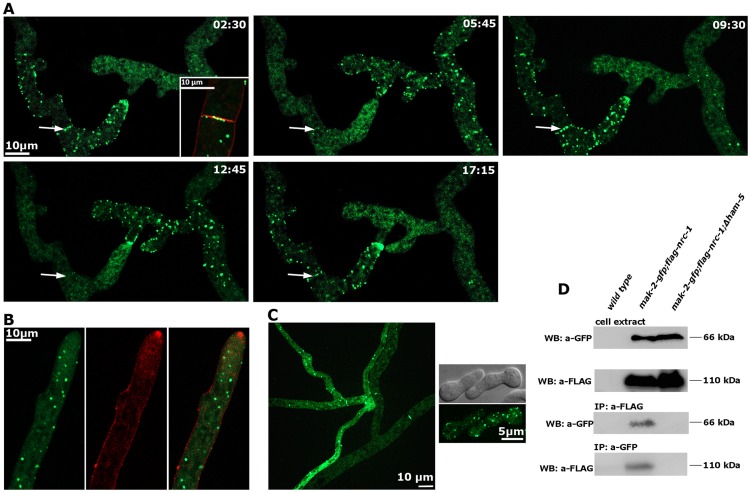
HAM-5 is a cell communication-specific scaffold of the MAK-2 module. (**A**) Dynamic HAM-5-GFP oscillation is observed in approaching tips within the established mycelium. Dynamic localization of HAM-5 to septa is indicated by arrows, and septum-associated HAM-5-GFP puncta are enlarged in the first image (insert co-labeled with FM4-64 to show plasma membrane and septum). See Video S5 for time course. (**B**) HAM-5 is not enriched at hyphal tips of non-communicating hyphae, although HAM-5-GFP spots are visible throughout the cytoplasm of the growing hypha. Plasma membrane and Spitzenkörper are labeled with FM4-64. (**C**) HAM-5-GFP puncta are also formed in Δ*mak-2* hyphae (left panel) and germlings (right panel), although the protein does not concentrate at germling tips. Note the distinct localization patterns of HAM-5-GFP in the two contacting hyphae in a Δ*mak-2* colony: multiple HAM-5 puncta are visible the approaching (left) hypha, while primarily the septal pores are labeled in the contacted (right) hypha and only few puncta are visible. (**D**) Reciprocal co-immunoprecipitation experiments of the tagged kinases flag-NRC-1 and MAK-2-GFP indicate interaction of the MAPK module in wild type but not in the Δ*ham-5* mutant.

Dynamic co-recruitment of HAM-5 and MAK-2 to communicating hyphal tips, septa and intracellular puncta was also observed in the mature colony (Figure 4 A, Figure S4). Moreover, HAM-5 and SOFT oscillated with opposing recruitment phases in communicating hyphae (Figure S4), indicating that the molecular machinery required for germling communication is also operating during hyphal anastomosis within the mature colony. In contrast to MAK-2 [17], HAM-5 was not detected at the apex of non-communicating hyphae (Figure 4 B). We therefore asked if MAK-2 activity is required for HAM-5 aggregation during cell signaling. When we localized HAM-5-GFP in Δ*mak-2* germlings, we observed the presence of HAM-5-GFP puncta, which remained stable over long time periods (Figure 4 C). Moreover, we noticed that intracolonial Δ*mak-2* hyphae that had (by chance) established physical contact grew in parallel over longer distances with HAM-5-GFP puncta formed primarily in one of the two hyphae, while septal pores were strongly labeled in the second cell (Figure 4 C). Quantification of this phenomenon revealed that 72±7% (n = 141) of the analyzed intracolonial hyphal pairs displayed this HAM-5 distribution. Thus, MAK-2 is essential to regulate the subcellular dynamics of HAM-5, but complex formation does not require the MAPK.

We reasoned that the interaction pattern of HAM-5 with multiple MAK-2 pathway components and its localization dynamics were consistent with a scaffold function of HAM-5 for the MAK-2 cascade. We tested this hypothesis by performing co-immunoprecipitation experiments (Figure 4 D). NRC-1 and MAK-2 co-precipitated when tagged versions of both proteins were co-expressed in wild type, while we were unable to detect interactions between the two kinases in a Δ*ham-5* background, indicating that HAM-5 is critical for maintaining the integrity of the kinase cascade.

### A network of Ste20-related kinases, RAS-2 and the adenylate cyclase capping protein CAP-1 signals upstream of the MAK-2 pathway

Our AP-MS analysis identified several proteins that specifically interacted with NRC-1, yet not MEK-2 or MAK-2 and thus could constitute upstream components of the MAK-2 pathway. Among them were the MAP4K STE-20, the small GTPase RAS-2/SMCO-7 and the capping protein CAP-1/NCU08008 of the adenylate cyclase (AC) complex (Table S1). A fully functional STE-20-GFP fusion construct (Figure S5) recovered STE-50, NRC-1, HAM-5 and RAS-2 in reciprocal AP-MS experiments (Figure 1 A), and Y2H assays confirmed physical interactions of STE-20 with HAM-5, NRC-1 and STE-50 (Figure 1 B, C; Figure S2). Moreover, NRC-1 and weakly also STE-50 interacted with RAS-2 through the Ras-association domains present in both proteins in Y2H tests. We did not detect any Y2H interaction of STE-50 and NRC-1 with the second Ras-type GTPase, RAS-1/NCU08823 (also called BAND; [46]), present in *N. crassa*, underscoring the specificity of the MS and Y2H analyses. We also detected MST-1, a recently identified accessory Ste20-related kinase of the septation initiation network, which localized to spindle pole bodies and septa [47], [48] as NRC-1- and MEK-2-interacting protein by AP-MS and Y2H analysis (Table S1; Figure 1 B). However, communication frequencies and MAK-2 activities of Δ*mst-1* and of a generated Δ*ste-20*;Δ*mst-1* double mutant indicated that MST-1 is not critically required for MAK-2 signaling (Table 1).

**Figure 5:**
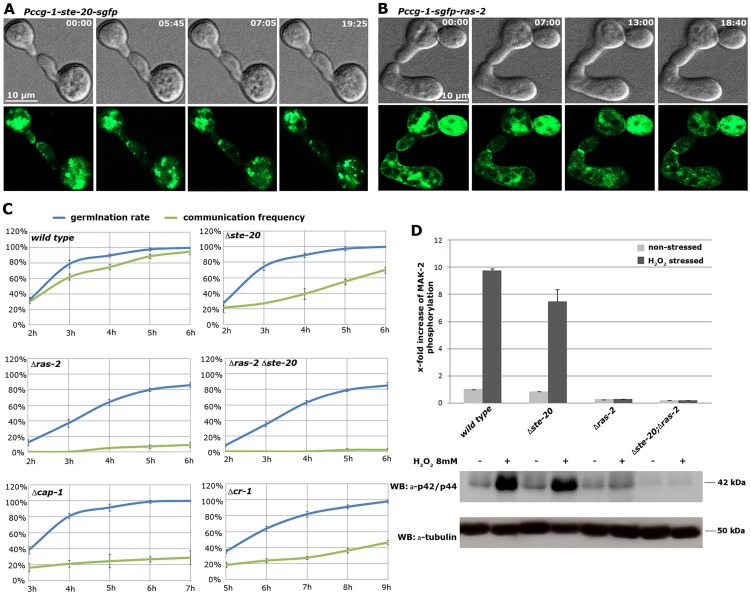
The MAK-2 pathway elements STE-20 and RAS-2 are important for cell-cell communication. (**A**) STE-20-GFP localizes in a stable manner to the apices of two communicating germlings and marks the site of contact. See Video S6 for time course. (**B**) GFP-RAS-2 associates with the entire plasma membrane of germinating spores and localizes at the contact point of two communicating cells. See Video S7 for time course. (**C**) Time course experiments to determine rates of germination and of chemotropic interactions of the indicated mutants. Note that due to delayed germination Δ*cr-1* and Δ*cap-1* strains are assayed at later time points than the other mutants. (**D**) Quantification of basal and stress-induced MAK-2 phosphorylation levels (detected with p42/44 antibodies) in cell extracts of exponentially growing liquid cultures of the indicated strains (n = 3). A representative Western blot is depicted below; tubulin was used as loading control.

Consistent with an involvement in cell-cell communication, we found that STE-20-GFP formed membrane-associated apical crescents in mature hyphae, was enriched in a stable manner at both communicating germling tips and localized at the contact point of interacting cells in addition to its association with septa in germlings as well as mature hyphae (Figure 5 A; Figure S5). Moreover, a functional GFP-RAS-2 construct distributed along the entire plasma membrane in germlings and hyphae and localized to the contact point of interacting germlings (Figure 5 B; Figure S5). As predicted for components that signal upstream of the MAK-2 cascade, we observed reduced MAK-2 activities and cell communication frequencies in Δ*ste-20*, in Δ*ras-2*, the previously described *ras-2* allele *smco-7* [49], and in two mutants affecting the predicted RAS-activating GDP-GTP-exchange factor CDC-25 (Table 1; Figure 5 C). Only residual levels of germling communication and MAK-2 activity were detected in a Δ*ste-20*;Δ*ras-2* double mutant, highlighting the joint importance of RAS-2 and STE-20 for self-signaling. The MAK-2 pathway is also induced by other external stimuli, such as reactive oxygen species [17], [28], and we asked if the identified proteins were also required for stress-induced activation of MAK-2. We determined that H_2_O_2_-induced activation of the MAK-2 pathway was abolished in Δ*ras-2* and in the Δ*ste-20*;Δ*ras-2* double mutant (Figure 5 D), indicating that both proteins are general components of the MAK-2 pathway.

We also tested for binding of these components to the AC capping protein CAP-1 and detected a positive Y2H interaction with STE-50, but not with any of the other proteins of the MAK-2 pathway (Figure 1 B). This may indicate that our AP-MS-based identification of CAP-1 in precipitates of STE-50, NRC-1 and STE-20 (Table S1; Figure 1 A) was mediated through STE-50. Nevertheless, we observed altered cell communication frequency and reduced MAK-2 activity in Δ*cap-1*, consistent with a functional involvement of CAP-1 in MAK-2 signaling (Table 1). Significantly, we also detected reduced tropic germling interactions in the AC mutant Δ*cr-1* (Figure 5 C), indicating that cAMP signaling is involved in, but is not essential for cell-cell communication. These data are consistent with a previous report, which showed that *cr-1* germlings are able to generate conidial anastomosis tubes, although no quantitative analysis was performed in this study [50].

## Discussion

**Figure 6:**
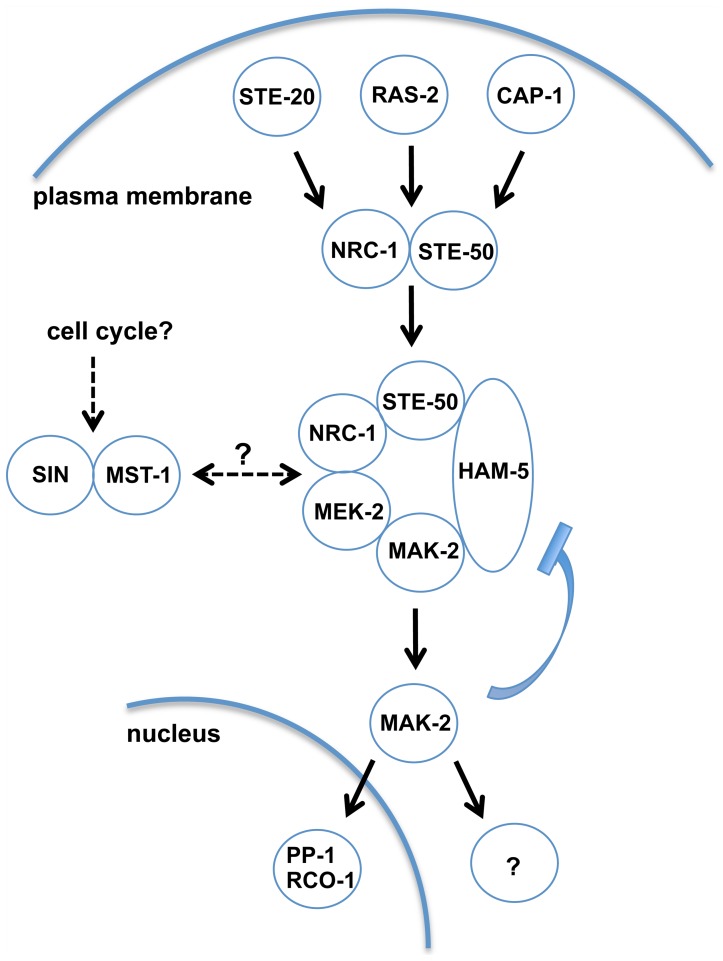
Model depicting network organization of the MAK-2 pathway and putative regulatory mechanisms. Ligand-induced activation of an unknown receptor may be transmitted through plasma membrane-associated STE-20, RAS-2 and CAP-1, which signal toward the NRC-1/STE-50 complex and recruit the MAPK cascade through activation and clustering of the scaffold HAM-5 at intracellular puncta. MAK-2 activation triggers nuclear gene expression through interaction with the transcription factor PP-1 and the RCO-1/RCM-1 complex and the cytosolic activation of the secretory pathway and cell polarity machineries to coordinate pulsed signal release and chemotrophic growth towards the partner cell, respectively. MAK-2 activity is also required for termination of the receiver phase, potentially through negative feedback phosphorylation of the MAPK and disassembly of the MAK-2/HAM-5 module. MAK-2 pathway function may also be regulated through the STRIPAK complex, the CK2 heterodimer, membrane lipid composition, the septum-associated septation initiation network SIN and motor protein-dependent vesicle trafficking.

Despite considerable progress in recent years, our mechanistic understanding of oscillatory MAK-2 behavior during homotypic cell communication is hampered by the fact that many components of the signal transduction machinery are still unknown and that the molecular functions of proteins known to be required for signaling are only poorly understood. One important finding of this study is the identification of STE-50 and HAM-5 as central components of the MAK-2 pathway (Figure 6). We propose STE-50 as tightly associated, regulatory subunit of NRC-1 and HAM-5 as scaffold protein of the MAK-2 cascade. Based on our data, STE-50 may have both NRC-1-activating as well as targeting functions, and we currently cannot rule out any of these hypotheses. Nevertheless, RAS-2 interacts with both NRC-1 and STE-50 in Y2H assays, and thus STE-50 may not be essential for membrane targeting, yet full complementation of Δ*ste-50* by expression of constitutive active NRC-1(P488S) indicates that STE-50 is critical for activation of the MAP3K. We do not have any evidence for a scaffold function of STE-50 in *N. crassa*, contrasting data obtained in other filamentous fungi, which have suggested interactions of STE-50 homologs with other kinases in addition to the MAP3K [40], [42], [51].

Central for our understanding of scaffold proteins is the archetypical MAP kinase scaffold Ste5p of the yeast mating pathway [52], [53]. However, Ste5p is restricted to budding yeast and close relatives [20], [54]. In contrast, homologs of HAM-5 are detected in all sequenced members of the Pezizomycotina subphylum (the group of filament-forming ascomycete fungi), but are absent from the genomes of unicellular ascomycete fungi (Figure S6). Thus, we propose HAM-5 as scaffold of the *N. crassa* MAK-2 cascade and of homologous MAPK modules in other filamentous fungi. This hypothesis is further strengthened by an accompanying study, which also identified HAM-5 as scaffold protein of the MAK-2 pathway [55]. We hypothesize that HAM-5 and MAK-2 are co-recruited to intracellular puncta in the presumed signal receiver phase [15], [16] and that some these puncta are subsequently targeted to the apical region of communicating cells. This process may potentially reflect the predicted auto-amplification of the incoming signal through the MAK-2 cascade or priming of the receiving cell for signal release during the next phase of communication [18]. The formation of HAM-5 puncta in the Δ*mak-2* mutant indicates that MAK-2 is not required for complex formation of HAM-5. HAM-5 puncta remain stable over long time periods in Δ*mak-2*, and hyphae in contact frequently display distinct HAM-5 localization patterns, suggesting that the two cells are locked into two distinct signaling modes. Thus, complex dispersion and the switch from signal receiving to signal sending require MAK-2 activity. One possible mechanism for complex disassembly and termination of the signal receiving phase of the cell may involve accumulating phosphorylation of the HAM-5 scaffold through MAK-2 and/or additional unknown kinases as proposed in [55]. This hypothesis is also in line with previous work reporting that unregulated (both reduced as well as increased) MAK-2 activity allows reasonable tip growth of vegetative hyphae, while cell-cell communication requires the regulated on/off switch of the MAK-2 cascade [17], [30]. In contrast to MAK-2 [17], HAM-5 did not accumulate at the apex of non-communicating hyphae, and residual MAK-2 pathway functionality is retained in Δ*ham-5* allowing reasonable rates of mycelial extension. These data imply a cell communication-specific function of HAM-5 for the MAK-2 cascade. In addition, previous work had identified the kinase adapter HYM-1/MO25, which associates with multiple Ste20-related kinases in *N. crassa* (this study; [17], [47]) and higher eukaryotes [56] as general platform that is required for MAK-2-dependent intercellular communication and for basic growth-associated functions of the MAK-2 pathway.

We identified STE-20 and RAS-2 that together are critical for signal input of the MAK-2 pathway. Moreover, the AC capping protein CAP-1 is also involved in cell-cell communication. The yeast homolog of CAP-1, Srv2p, was identified as part of a RAS-responsive AC complex in *S. cerevisiae* [57], [58]. CAP-1 homologs also play a critical role in regulating actin dynamics and cell polarity in various fungi as well as higher eukaryotes [59]–[62]. Thus, the association of STE-50 with CAP-1 may link upstream components of the MAK-2 pathway with Ras/cAMP signaling and with cell morphogenesis through regulation of actin dynamics. We did not detect the second STE-20-related kinase CLA-4 as MAK-2 pathway-associated protein in our proteomics analysis, consistent with previous Y2H data that indicate no physical interaction between NRC-1 and CLA-4 [17]. Thus, CLA-4, which was recently implicated in self-signaling in *N. crassa* [63], may function as part of another module, such as the predicted BEM-1/CDC-42/RAC/CDC-24/CLA-4 complex that regulates cell polarity and potentially also chemotrophic growth [64]-[66]. Intriguingly, we also identified the Ste20-related kinase MST-1 as NRC-1- and MEK-2-interacting protein (this study; [47]). Although the significance of these interactions is currently unclear, the entire MAK-2 cascade including STE-50 and HAM-5 associates with septa ([16], [17]; this study). The dynamic localization of HAM-5 at septa during intra-colony communication and its strong septal pore association may therefore indicate that MAK-2-dependent signals can originate from septa and/or that incoming signals are integrated by the MAPK pathway at these sites. This speculation is supported by accumulating evidence of central functions of septal pores as signaling hubs within the fungal colony [67]–[69].

Finally, the association of the casein kinase 2 holoenzyme and the PP2A heterotrimer with the MAK-2 cascade opens the intriguing possibility for activity regulation of the MAPK cascade. Analogous results were obtained for the mammalian casein kinase 2, which also associates with phosphatase 2A and down-regulates the PP2A substrate MEK1 [70], [71]. A similar, regulatory role may also be attributed to the recently defined STRIPAK complex [27], [28], [72]. Significantly, we did not detect the STRIPAK subunits HAM-2 and HAM-4 in our MS analysis, and thus only a sub-complex consisting of the PP2A heterotrimer and the kinase adaptor protein MOB-3 may associate with and regulate the MAK-2 cascade. MAK-2 pathway regulation may also occur through modulation of lipid composition and thus membrane dynamics, which is central for the organization and dynamic localization of multiple signal transduction pathways, including the yeast pheromone pathway [73], and has also been implicated in self-signaling in *N. crassa* [74]. Yeast homologs of the *N. crassa* kinases YPK-1 and NRC-2 function as regulators of so-called flippase complexes [75], [76], which are primarily localized to the plasma membrane at sites of polarized growth, and phospholipid flipping has been shown to regulate Cdc42p signaling during polarized growth in yeast [77]. Because *N. crassa ypk-1* and *nrc-2* strongly phenocopy vegetative and developmental traits of MAK-2 pathway mutants [78], [79], the association of YPK-1 with NRC-1 and the cell communication defects detected in deletion and temperature-sensitive *ypk-1* mutants will be of particular interest for dissecting oscillatory MAK-2 signaling and the chemotropic behavior of communicating cells.

## Materials and Methods

### Strains, media and tagged protein constructs

Strains and oligonucleotides used in this study are listed in Tables S2 and S3, respectively. General genetic procedures and media used in the handling of *N. crassa* are available through the Fungal Genetic Stock Center (www.fgsc.net). Fusion proteins were ectopically expressed under the control of the *ccg-1* or *gpd-1* promoters at the *his-3* locus. The ORFs of *ste-50, ham-5, ste-20 and ras-2* were amplified by PCR as annotated by the *N. crassa* database and introduced into pMF272, pNGFP and pJV15-2 [80]–[82]. For co-expression of tagged proteins, the *pccg-1-sgfp* sequence of the vector pNGFP [81] was replaced with the inversely oriented *ccg-1* and *gpd-1* promoters amplified from the vector pBiFC [83] to generate the vector pCCG1-pGPD1, which allowed insertion of fusion-constructs via SgsI/SpeI (*mak-2-sgfp*) and SwaI/EcoRI (*3xflag-nrc-1*) amplified from the template plasmids pMF272-mak2 and pFLAG-nrc1 [17]. Resulting plasmids were transformed into *his-3* and/or *his-3;*Δ strains. Complementation of tropic interaction, growth and developmental defects of the deletion strain was used to confirm functionality of the constructs.

### Protein methods

Co-immunoprecipitation experiments were performed as described [28], [84]. Conditions and plasmids used for the Y2H assays are specified in [83], [85]. Basal and stress-induced MAK-2 activity of exponentially growing *N. crassa* liquid cultures was determined using polyclonal rabbit α-Phospho-p44/42 MAPK (Cell Signaling Technology, USA) and goat α-rabbit IgG-HRP (Santa Cruz, USA) as primary and secondary antibodies, respectively as described [86], [87]. Briefly, exponentially growing, liquid cultures were harvested gently by filtration using a Büchner funnel and ground in liquid nitrogen. The frozen mycelial powder was incubated in 95% ethanol at -20°C for ≥12 h, the supernatant removed after centrifugation and the pellet vacuum-dried in a SpeedVac concentrator (Thermo Fisher Scientific, USA). extraction buffer (50 mM Tris/HCL pH 7,5, 100 mM KCl, 10 mM MgCl_2_, 0.15% NP-40, 5 mM NaF, 1 mM PEFA, 1 mM Na_3_VO_4_, 25 mM β-glycerophosphate, 2 mM benzamidine, 2 ng/µl pepstatin A, 10 ng/µl aprotinin, 10 ng/µl leupeptin) was added, the samples mixed and incubated at 80°C for 5 min and the supernatant collected after centrifugation. After a second round of extraction, the supernatants pooled, subjected to another centrifugation step, and the protein concentration determined using a Nanodrop spectrophotometer (ND-1000, Peqlab, Germany). Sample volumes corresponding to 75 µg total protein per lane were subjected to SDS polyacrylamide gel electrophoresis. ≥3 biological replicates were quantified for each experiment. For quantification of MAK-2 phosphorylation levels, exposed films were scanned at a resolution of 600 dpi and densitometry was performed on the resulting tif files employing the AIDA Image Analyzer (version 4.22; raytest Isotopenmessgeräte, Germany) in transmission mode. Intensity values [arbitrary units] measured within a region of interest of fixed size containing the MAK-2 protein bands were corrected by subtraction of local background, normalized to the protein amount loaded and used for further evaluation.

GFP-trap experiments, mass spectrometry and database analysis were performed as described [28], [88]. Pulverized mycelium was mixed 1∶1 with extraction buffer and centrifuged (1 h, 10,000 rpm, Sorvall SS34 rotor) in order to obtain crude cell extracts. Cell extracts were incubated with 2 µl GFP-trap beads (Chromotek, Germany) per 15 ml cell extract on a rotator for 2 h at 4°C. The beads were washed three times with IP buffer, associated proteins were recovered by boiling them in Laemmli buffer and separated by SDS polyacrylamide gel electrophoresis analysis. Peptides of in-gel trypsinated proteins were extracted from Coomassie-stained gel slices. Peptides of 5 µl sample solution were trapped and washed with 0.05% trifluoroacetic acid on an Acclaim PepMap 100 column (75 µm×2 cm, C18, 3 µm, 100 Å, P/N164535 Thermo Scientific, USA) at a flow rate of 4 µl/min for 12 min. Analytical peptide separation by reverse phase chromatography was performed on an Acclaim PepMap RSLC column (75 µm×15 cm, C18, 3 µm, 100 Å, P/N164534 Thermo Scientific, USA) running a gradient from 96% solvent A (0.1% formic acid) and 4% solvent B (acetonitrile, 0.1% formic acid) to 50% solvent B within 25 min at a flow rate of 250 nl/min. Peptides eluting from the chromatographic column were on-line ionized by nano-electrospray using the Nanospray Flex Ion Source (Thermo Scientific, USA) and transferred into the mass spectrometer. Full scans within m/z 300–1850 were recorded by the Orbitrap-FT analyzer at a resolution of 60,000 at m/z 400. Each sample was analyzed using two different fragmentation techniques applying a data-dependent top 5 experiment: collision-induced decay with multistage activation and readout in the LTQ Velos Pro linear ion trap, and higher energy collision dissociation and subsequent readout in the Orbitrap-FT analyzer. LC/MS method programming and data acquisition was performed with XCalibur 2.2 (Thermo Scientific, USA). Orbitrap raw files were analyzed with Proteome Discoverer 1.3 (Thermo Scientific, USA) using the Mascot and Sequest search engines against the *N. crasssa* protein database with the following criteria: peptide mass tolerance 10 ppm, MS/MS ion mass tolerance 0.8 Da, and up to two missed cleavages allowed.

### Microscopy

Spinning disc confocal microscopy was performed as described [47] using an inverted Axio Observer Z1 microscope (Zeiss, Germany) equipped with a CSU-22 confocal scanner unit and a CCD camera (Axiocam MRm Rev.3). ZEN Blue 2012 software (Zeiss, Germany) was used for image/video acquisition and image analysis. Plasma membrane was stained with FM4-64 (1 mg/ml-1), and time-lapse imaging was performed at capture intervals of 20 s for periods up to 40 min using a C-Apochromat 63x/1.2 W objective. Image series were converted into movies (*.mov).

Tropic germling interactions of ≥100 germlings in each of 2-3 biological replicates per experiment were quantified as described [28], [65]. In brief, strains were grown on Vogel's minimal media slants for 7 days at 26°C. Conidia were harvested with 1 to 2 ml H_2_O, and the conidial suspension was filtered through cheesecloth. A total number of 5×10^6^ fresh spores were spread out on a minimal media plate, incubated at 30°C and analyzed after the indicated time points using a Zeiss Axiophot 2 microscope with a Zeiss Plan-Apochromat 63x/1.40 oil immersion objective. Only germlings that had produced germ-tubes of at least 2 µm length and were localized within a distance smaller than 10 µm to another germling were included in the trophic interaction analysis.

## Supporting Information

Figure S1 Mutant characteristics of MAK-2 pathway components. (**A**) Pictures of communicating germlings of the indicated strains were taken after 4-6 h germination at 30°C. (**B**) MAK-2 phosphorylation levels were determined with p42/44 antibodies in cell extracts of exponentially growing liquid cultures of the indicated strains. Tubulin was used as loading control.
(PDF)

Figure S2 Interaction network of the MAK-2 pathway. (**A**) Physical interactions between MAK-2 pathway components were mapped in yeast two-hybrid (Y2H) tests. The indicated constructs were co-expressed in strain AH109 and yeast growth was analyzed on the indicated selective media. (**B**) Reciprocal co-immunoprecipitation experiments from cell extracts co-expressing the functionally tagged proteins NRC-1, MEK-2 and MAK-2 and HAM-5 indicate interaction of all three kinases with HAM-5.
(PDF)

Figure S3 Comparative characterization of Δ*ste-50* and Δ*ham-5*. Analysis of macroscopic appearance (**A**), mycelial extension rate (**B**), sexual development (**C**), germling communication frequency (**D**), and basal/stress-stimulated MAK-2 activity level (**E**) indicates that Δ*ste-50* fully phenocopies Δ*mak-2* defects, while Δ*ham-5* retains residual MAK-2 pathway functionality.
(PDF)

Figure S4 Co-localization of HAM-5 with the SOFT/MAK-2 cell communication machinery in communicating hyphae within the established mycelium. (**A**) MAK-2-GFP and HAM-5-mCherry co-localize in a dynamic manner to opposing tips of two communicating hyphae. Note the dynamic nuclear accumulation of MAK-2, yet not HAM-5 in the presumed signal receiver phase. (**B**) Dynamic recruitment to communicating hyphal tips is also observed for SOFT. (**C**) HAM-5-mCherry and SO-1-GFP oscillate with strictly opposing recruitment phases in communicating hyphae and do not co-localize.
(PDF)

Figure S5 Characterization of *ste-20* (left) and *ras-2* (right panels). (**A**) Macroscopic appearance and mycelial extension rates of the indicated deletion mutants and complemented strains. (**B**) Localization of GFP fusion constructs in mature hyphae. See Videos S8 and S9 for time courses. Plasma membrane and Spitzenkörper are labeled with FM4-64. H1-RFP was used to label nuclei (left panel only).
(PDF)

Figure S6 Phylogram of fungal HAM-5 homologs. The tree was generated by using ClustalX 2.1 with bootstrap support (111 random number generator seed and 1000 bootstrap trials) and predicted protein sequences from selected ascomycete proteins. Note that only the WD40 domains of each protein were included in this analysis in order to use the WD40 domain of *N. crassa* HAM-3 as outgroup member (similar tree topologies were obtained with full length sequences; multiple alignment parameters: gap opening 10.0, gap extension 0,2, delay divergent sequences 30%, protein weight matrix gonnet series).
(PDF)

Table S1 AP-MS analysis of the MAK-2 pathway.
(XLSX)

Table S2 *N. crassa* strains used in this study.
(DOCX)

Table S3 Oligonucleotides used in this study.
(DOCX)

Video S1 Time-course of GFP-STE-50 localization during germling communication and chemotrophic growth. GFP-STE-50 is recruited in an oscillatory manner to the tips of communicating germlings.
(MOV)

Video S2 Time-course of HAM-5-GFP localization during germling communication and chemotrophic growth. HAM-5-GFP is recruited in an oscillatory manner to the tips of communicating germlings.
(MOV)

Video S3 Time-course of HAM-5-mCHERRY and MAK-2-GFP co-localization during germling communication and chemotrophic growth. Both proteins are co-recruited in an oscillatory manner to the tips of communicating germlings.
(MOV)

Video S4 Time-course of HAM-5 puncta formation at the nuclear envelope during germling communication. HAM-5 and histone H1 are tagged with GFP and RFP, respectively.
(MOV)

Video S5 Time-course of HAM-5-GFP localization in the established colony. HAM-5-GFP is recruited in an oscillatory manner to the tips and septa of communicating hyphae.
(MOV)

Video S6 Time-course of STE-20-GFP localization during germling communication and chemotrophic growth. STE-20-GFP is enriched at both approaching tips and accumulates at the contact point of the two cells.
(MOV)

Video S7 Time-course of GFP-RAS-2 localization during germling communication and chemotrophic growth. GFP-RAS-2 associates with the plasma membrane and strongly accumulates at the contact point of the two cells.
(MOV)

Video S8 Time-course of STE-20-GFP localization at the tip of mature hyphae. STE-20-GFP form a dynamic apical crescent and subapical collar at the hyphal tip.
(MOV)

Video S9 Time-course of GFP-RAS-2 localization at the tip of mature hyphae. GFP-RAS-2 associates with the entire plasma membrane in a non-specific manner in established hyphae.
(MOV)
