# Supplementary figures and images for: Fungal Communication Requires the MAK-2 Pathway Elements STE-20 and RAS-2, the NRC-1 Adapter STE-50 and the MAP Kinase Scaffold HAM-5
Source: PLoS Genet. 2014 Nov 20;10(11):e1004762. doi: 10.1371/journal.pgen.1004762 (PMC4239118; doi:10.1371/journal.pgen.1004762)

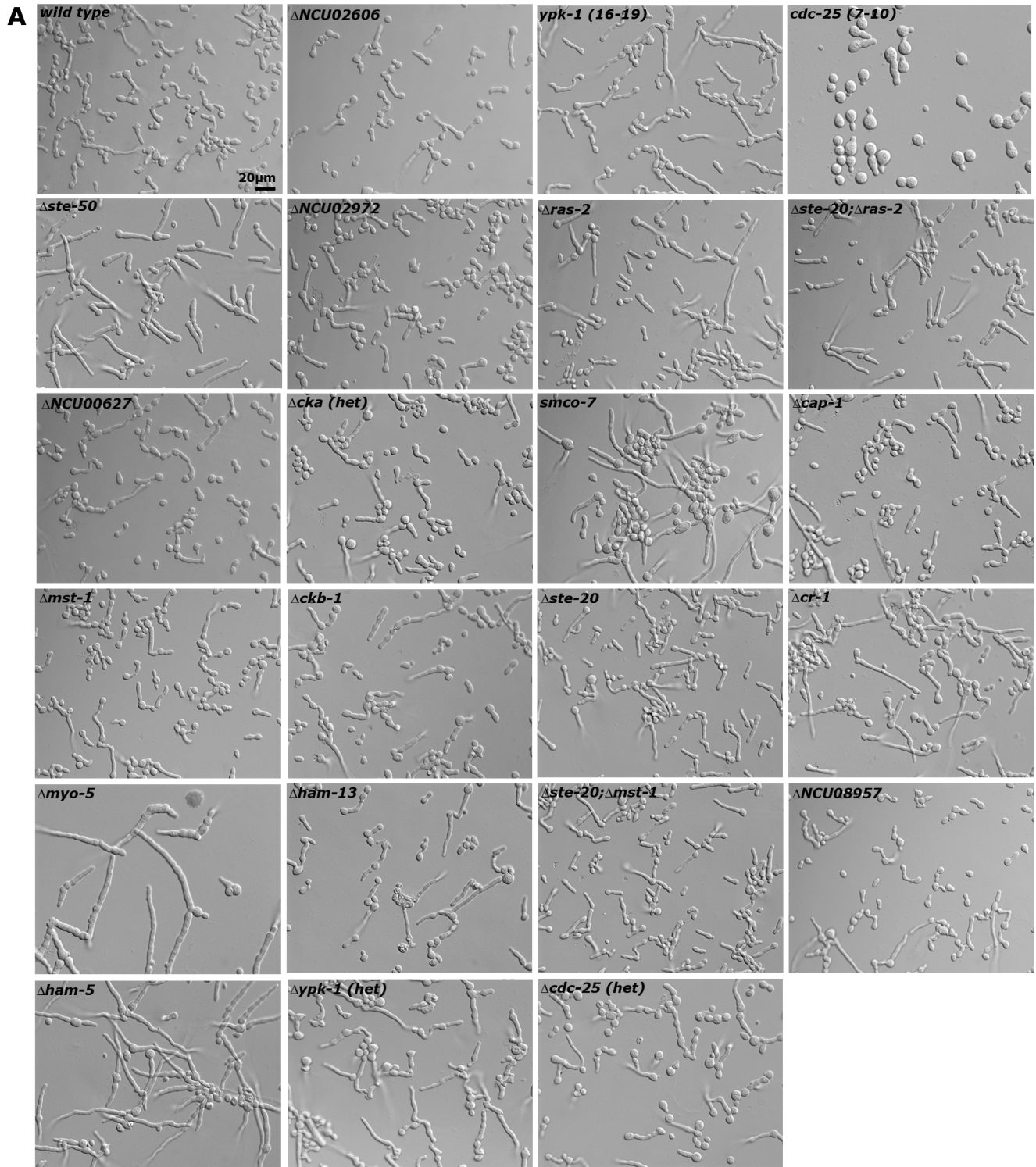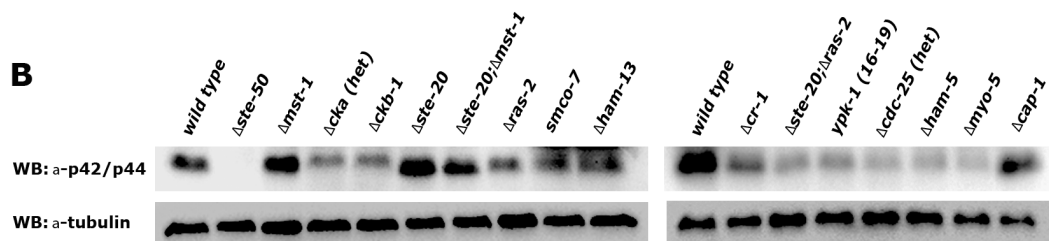

Supplement: Figure S1 — Mutant characteristics of MAK-2 pathway components. (A) Pictures of communicating germlings of the indicated strains were taken after 4-6 h germination at 30°C. (B) MAK-2 phosphorylation levels were determined with p42/44 antibodies in cell extracts of exponentially growing liquid cultures of the indicated strains. Tubulin was used as loading control. (PDF) [file pgen.1004762.s001.pdf]

A

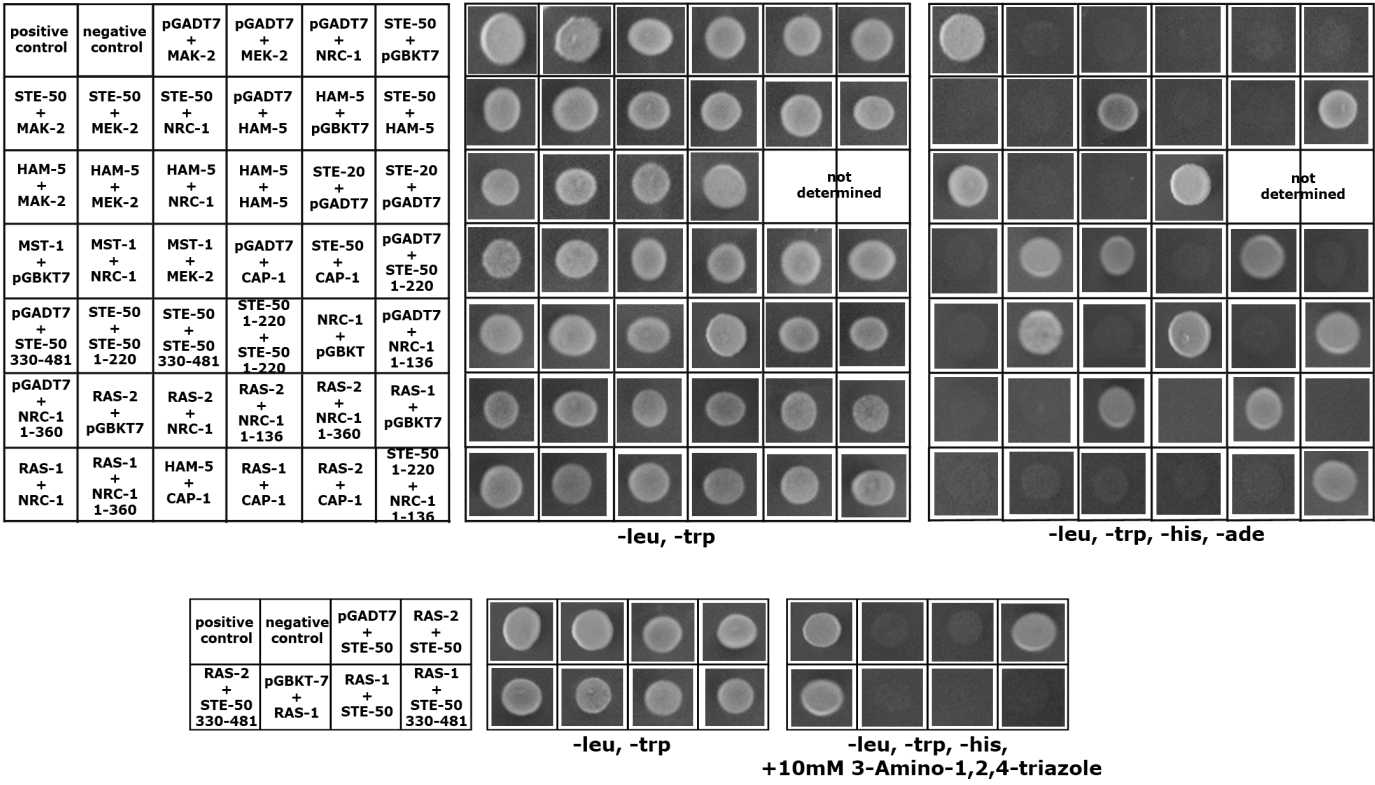

B

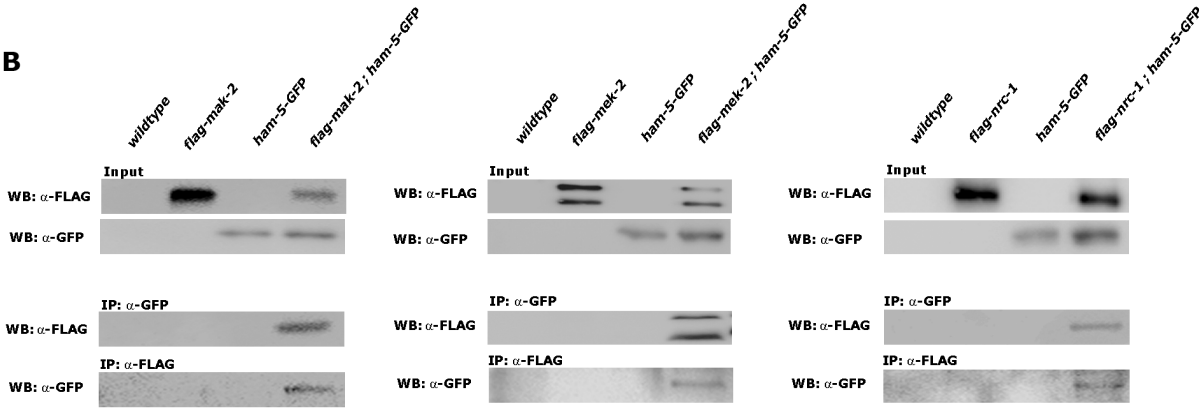

Supplement: Figure S2 — Interaction network of the MAK-2 pathway. (A) Physical interactions between MAK-2 pathway components were mapped in yeast two-hybrid (Y2H) tests. The indicated constructs were co-expressed in strain AH109 and yeast growth was analyzed on the indicated selective media. (B) Reciprocal co-immunoprecipitation experiments from cell extracts co-expressing the functionally tagged proteins NRC-1, MEK-2 and MAK-2 and HAM-5 indicate interaction of all three kinases with HAM-5. (PDF) [file pgen.1004762.s002.pdf]

**A**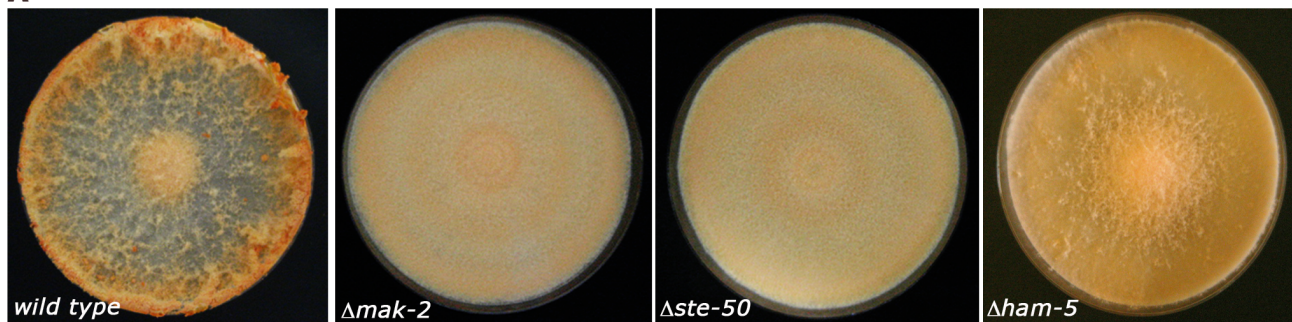**B**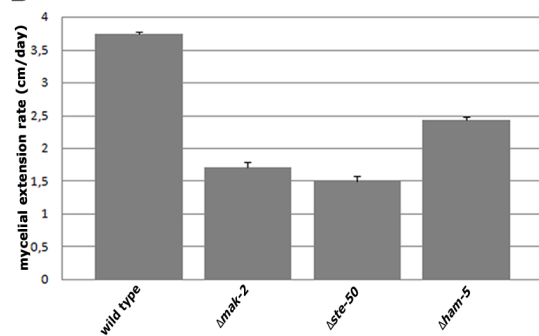**C**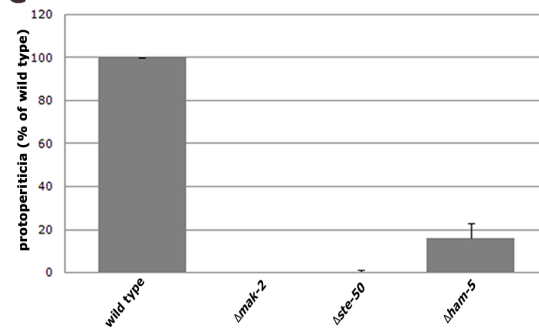**D**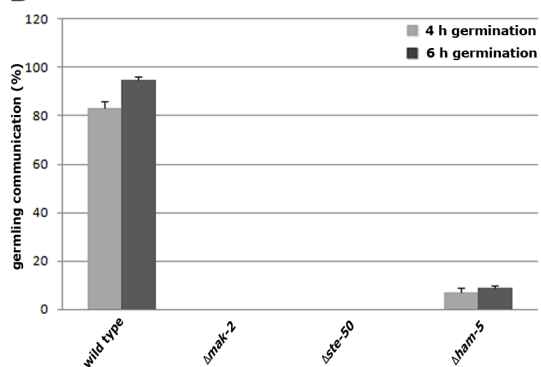**E**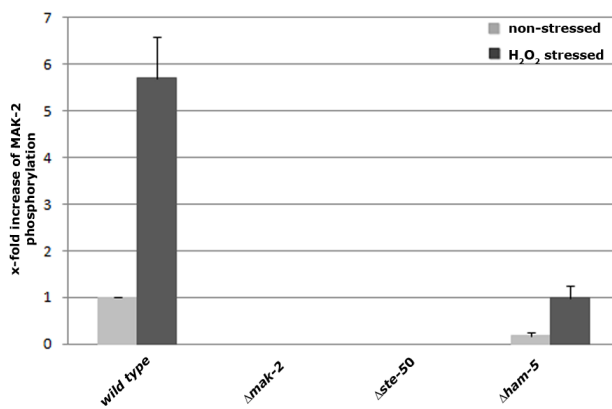

Supplement: Figure S3 — Comparative characterization of Δste-50 and Δham-5. Analysis of macroscopic appearance (A), mycelial extension rate (B), sexual development (C), germling communication frequency (D), and basal/stress-stimulated MAK-2 activity level (E) indicates that Δste-50 fully phenocopies Δmak-2 defects, while Δham-5 retains residual MAK-2 pathway functionality. (PDF) [file pgen.1004762.s003.pdf]

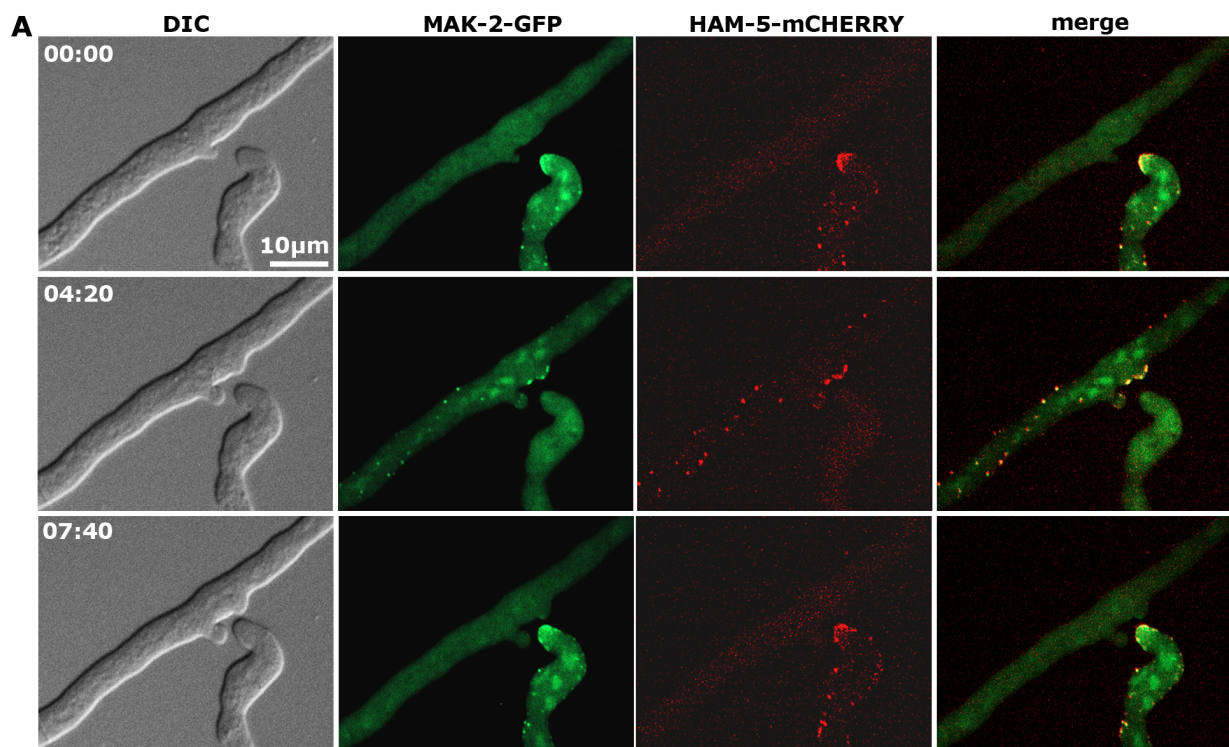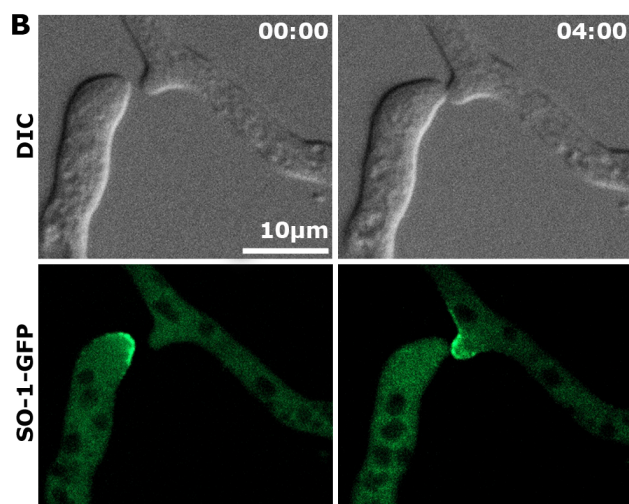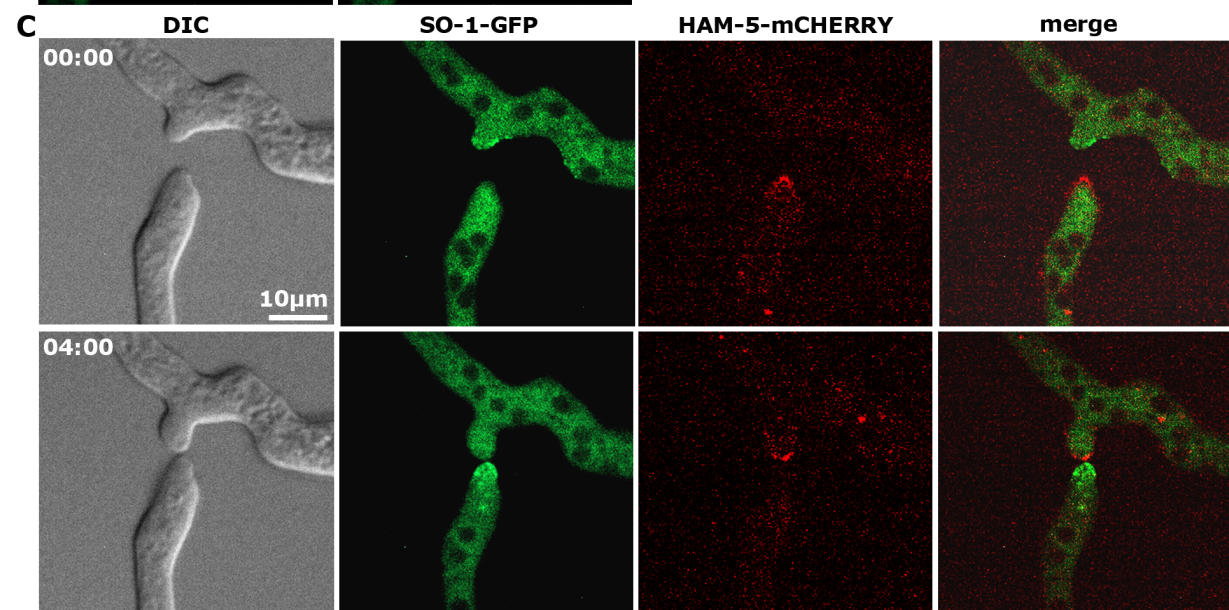

Supplement: Figure S4 — Co-localization of HAM-5 with the SOFT/MAK-2 cell communication machinery in communicating hyphae within the established mycelium. (A) MAK-2-GFP and HAM-5-mCherry co-localize in a dynamic manner to opposing tips of two communicating hyphae. Note the dynamic nuclear accumulation of MAK-2, yet not HAM-5 in the presumed signal receiver phase. (B) Dynamic recruitment to communicating hyphal tips is also observed for SOFT. (C) HAM-5-mCherry and SO-1-GFP oscillate with strictly opposing recruitment phases in communicating hyphae and do not co-localize. (PDF) [file pgen.1004762.s004.pdf]

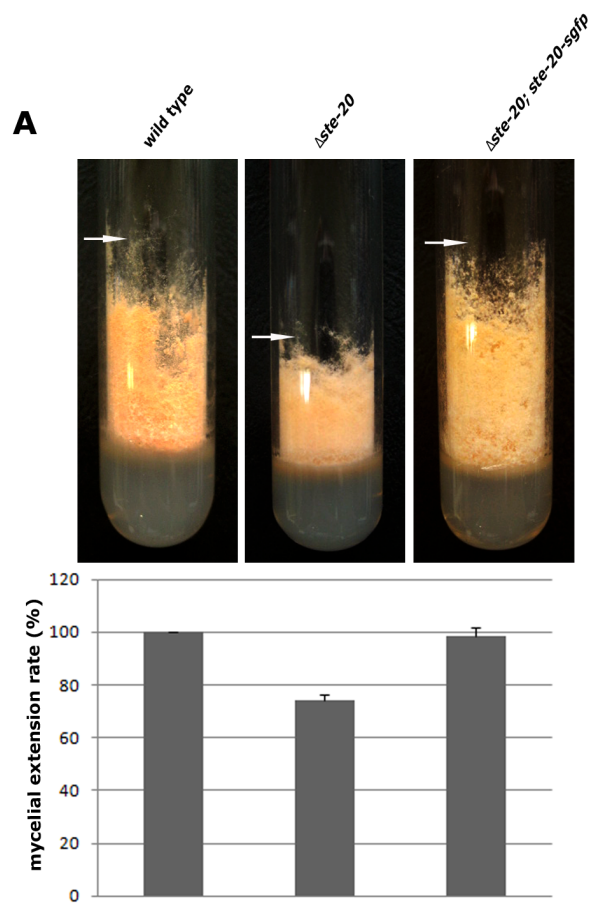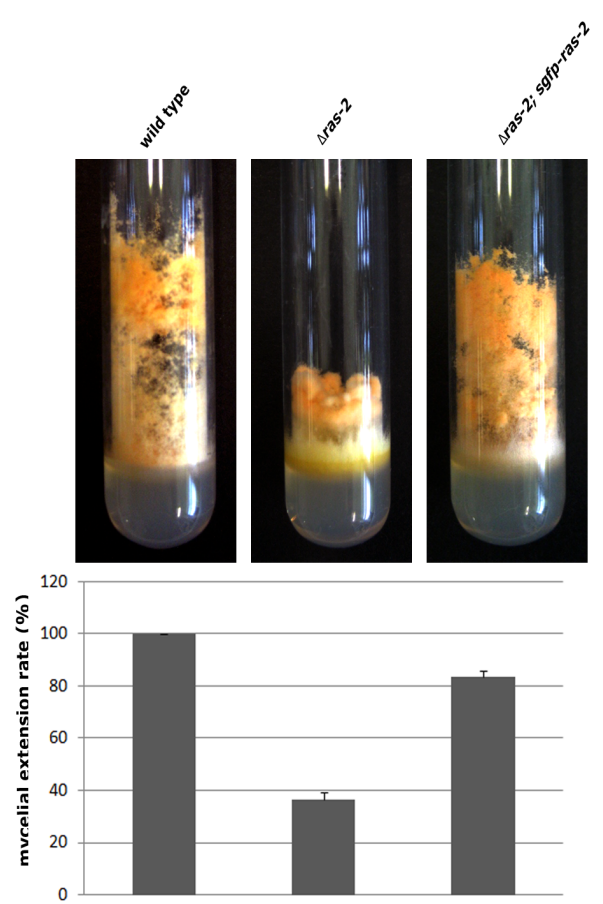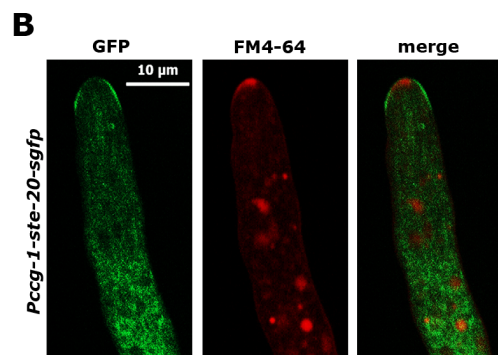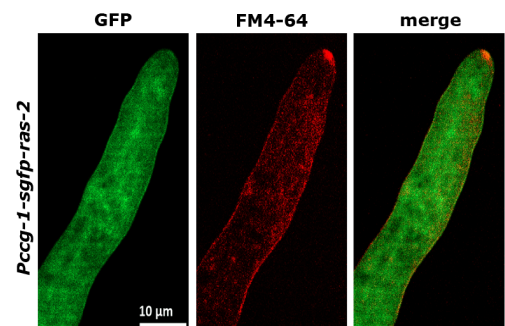

Supplement: Figure S5 — Characterization of ste-20 (left) and ras-2 (right panels). (A) Macroscopic appearance and mycelial extension rates of the indicated deletion mutants and complemented strains. (B) Localization of GFP fusion constructs in mature hyphae. See Videos S8 and S9 for time courses. Plasma membrane and Spitzenkörper are labeled with FM4-64. H1-RFP was used to label nuclei (left panel only). (PDF) [file pgen.1004762.s005.pdf]

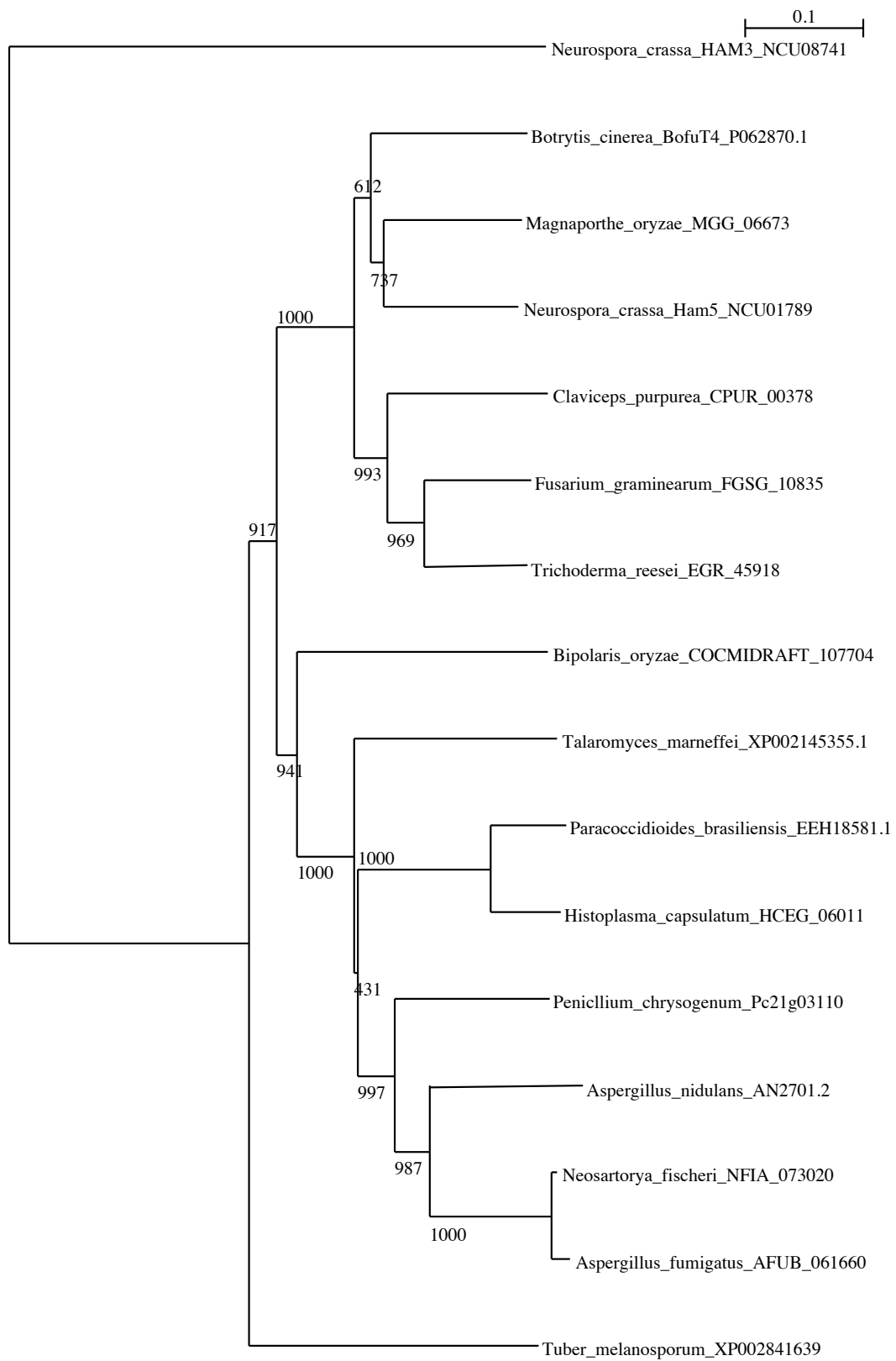

Supplement: Figure S6 — Phylogram of fungal HAM-5 homologs. The tree was generated by using ClustalX 2.1 with bootstrap support (111 random number generator seed and 1000 bootstrap trials) and predicted protein sequences from selected ascomycete proteins. Note that only the WD40 domains of each protein were included in this analysis in order to use the WD40 domain of N. crassa HAM-3 as outgroup member (similar tree topologies were obtained with full length sequences; multiple alignment parameters: gap opening 10.0, gap extension 0,2, delay divergent sequences 30%, protein weight matrix gonnet series). (PDF) [file pgen.1004762.s006.pdf]
