# Supplementary material for: Fungal Communication Requires the MAK-2 Pathway Elements STE-20 and RAS-2, the NRC-1 Adapter STE-50 and the MAP Kinase Scaffold HAM-5
Source: PLoS Genet. 2014 Nov 20;10(11):e1004762. doi: 10.1371/journal.pgen.1004762 (PMC4239118; doi:10.1371/journal.pgen.1004762)
Supplement: Table S2 — N. crassa strains used in this study. (DOCX) [file pgen.1004762.s008.docx]

**Table S2. *N. crassa* strains used in this study**

| **Strains** | **Genotype** | **Source** |
| --- | --- | --- |
| *wild type 74* | *OR231 Mat A* | FGSC #987 |
| *wild type ORS* | *SL6 Mat a* | FGSC #4200 |
| *his-3 A* | *his-3 Mat A* | FGSC #6103 |
| *his-3 a* | *his-3 Mat a* | FGSC #718 |
| *trp-1;his-3* | *trp-1- his-3-* | [[25](#_ENREF_25)] |
| *nic-3;his-3* | *nic-3- his-3-* | [[25](#_ENREF_25)] |
| *∆ste-50* | *hph::ste-50∆* | FGSC #17041 x FGSC #4200 |
| *∆ste-50;his-3* | *hph::ste-50∆ his-3-* | FGSC #17041 x FGSC #718 |
| *∆ham-5* | *hph::ham-5∆* | FGSC #15045 |
| *∆ham-5;his-3* | *hph::ham-5∆ his-3-* | FGSC #15045 x FGSC #6103 |
| *∆myo-V* | *hph::myo-V∆* | FGSC #11422 |
| *∆cka* | *hph::cka∆ + cka^+^ mus-51∆::bar^R^* | FGSC #17973 |
| *∆ckb-1* | *hph::NCU02972∆* | FGSC #15567 |
| *∆ypk-1(het)* | *hph::ypk-1∆ + ypk^+^ mus-52∆::bar^R^* | FGSC #13416 |
| *ypk-1(16-19)* |  | [[79](#_ENREF_79)] |
| *∆NCU00627* | *hph::NCU00627∆* | FGSC #16766 |
| *∆NCU02606* | *hph::NCU02606∆* | FGSC #17297 |
| *∆NCU02972* | *hph::NCU02972∆* | FGSC #19297 |
| *∆NCU06265* | *hph::NCU06265∆* | FGSC #11245 |
| *∆NCU08957* | *hph::NCU08957∆* | FGSC #19203 |
| *∆mst-1* | *hph::mst-1∆* | [[47](#_ENREF_47)] |
| *∆ste-20* | *hph::ste-20∆* | FGSC #11325 |
| *∆ste-20;his-3* | *hph::ste-20∆ his-3-* | FGSC #11325 x FGSC #6103 |
| *∆mst-1;∆ste-20* | *hph::mst-1∆ hph::ste-20∆* | This study |
| *∆ras-2* | *hph::ras-2∆* | FGSC #12467 |
| *∆ras-2;his-3* | *hph::ras-2∆ his-3-* | FGSC #12467 x FGSC #6103 |
| *∆ras-2;∆ste-20* | *hph::ras-2∆; hph::ste-20∆* | This study |
| *smco-7* |  | [[49](#_ENREF_49)] |
| *∆cdc-25* | *hph::cdc-25∆ + cdc-25^+^ mus-51∆::bar^R^* | FGSC #16014 |
| *cdc-25(7-10)* |  | [[79](#_ENREF_79)] |
| *∆cap-1* | *hph::cap-1∆* | FGSC #12371 |
| *∆cr-1* | *hph::cr-1∆* | FGSC #11514 x FGSC #987 |
| *∆mak-2* | *hph::mak-2∆* | [[17](#_ENREF_17)] |
| *∆mak-2;his-3* | *hph::mak-2∆ his-3* | This study |
| *∆mak-2:ham-5-gfp* | *hph::mak-2∆ Pccg-1-ham-5-gfp:: his-3* | This study |
| *sgfp-ste50* | *Pccg-1-sgfp-ste-50::his-3 hph::ste-50∆* | This study |
| *ham-5-sgfp* | *Pccg-1-ham-5-sgfp::his-3 hph::ham-5∆* | This study |
| *ste-20-sgfp* | *Pccg-1-ste-20-sgfp::his-3 hph::ste-20∆* | This study |
| *sgfp-ras-2* | *Pccg-1-sgfp-ras-2::his-3 hph::ras-2∆* | This study |
| *mak-2-sgfp* | *Pccg-1-mak-2-sgfp::his-3 hph::mak-2∆* | [[17](#_ENREF_17)] |
| *flag-nrc-1;∆ste-50* | *Pccg-1-3xflag-nrc-1::his-3 hph::ste-50∆* | This study |
| *flag-nrc-1(P488S);∆ste-50* | *Pccg-1-3xflag-nrc-1(P488S)::his-3 hph::ste-50∆* | This study |
| *mak-2-sgfp;flag-nrc-1* | *Pccg-1-mak-2-sgfp-Pgpd-1-3xflag-nrc-1::his-3* | This study |
| *mak-2-sgfp;flag-nrc-1;∆ham-5* | *Pccg-1-mak-2-sgfp-Pgpd-1-3xflag-nrc-1::his-3 hph: ham-5∆* | This study |
| *flag-ste-7;trp-1* | *Pccg-1-3xflag-ste-7::his-3 trp-1-* | [[17](#_ENREF_17)] |
| *flag-nrc-1;trp-1* | *Pccg-1-3xflag-nrc-1::his-3 trp-1-* | [[17](#_ENREF_17)] |
| *flag-mak-2;trp-1* | *Pccg-1-3xflag-mak-2::his-3 trp-1-* | This study |
| *ham-5-sgfp;nic-3* | *Pccg-1-ham-5-sgfp::his-3 nic-3-* | This study |
| *soft-1-sgfp* | *Pccg-1-soft-1-sgfp::his-3* | [[89](#_ENREF_89)] |

89. Fleissner A, Glass NL (2007) SO, a protein involved in hyphal fusion in *Neurospora crassa*, localizes to septal plugs. Eukaryot Cell 6: 84-94.
