# Supplementary material for: Fungal Communication Requires the MAK-2 Pathway Elements STE-20 and RAS-2, the NRC-1 Adapter STE-50 and the MAP Kinase Scaffold HAM-5
Source: PLoS Genet. 2014 Nov 20;10(11):e1004762. doi: 10.1371/journal.pgen.1004762 (PMC4239118; doi:10.1371/journal.pgen.1004762)
Supplement: Table S3 — Oligonucleotides used in this study. (DOCX) [file pgen.1004762.s009.docx]

**Table S3. Oligonucleotides used in this study**

| **Name** | **Sequence 5‘- 3‘** |
| --- | --- |
| GFP-fusion constructs: |  |
| AD1785_XbaI5 | **tct aga** ATG TCG GTC CCC GG |
| AD1785_PacI3 | **tta att aa**G ATC ATC TCA CTA TGA TG |
| AD_gfpras2_AscI5f | **ggc gcg cc**C ATG GTG GGG AAC |
| AD_gfpras2_PacI3r | **tta att aa**T CAT ATA ATC AAA CAC TTT |
| AD3894_GFP_SmaI5 | **ccc ggg** CAT GGA CGG CCA G |
| AD3894_GFP_PacI3 | **tta att aa**C TGC CCT TTA CGG GC |
| SL_0455forAscI | **ggc gcg cc**A ATG AAC TTT GAC TCG GGC ACC |
| SL_0455revPacI | **tta att aa**C TAT ATG ATA CCT CCC GGT GG |
|  |  |
| Co-expression constructs: |  |
| AD_double_ccg1_SgsI | **ggc gcg cc**T TTG GTT GAT GTG AGG GGT TG |
| AD_double_gpd1_EcoRI | **gaa ttc** GGA TCC CGG GAT TTA AAT TGA TGT CTG C |
| AD_mak2gfp_SgsI5: | **ggc gcg ccc** ATG AGC AGC GCA C |
| AD_mak2gfp_SpeI3 | **act agt** TTA CTT GTA CAG CTC |
| AD_nrc1flag_SwaI5 | **att taa at**A TGG ACT ACA AAG |
| AD_mek2flag_EcoRI3 | **gaa ttc** TCA AAA TCG GCC AG |
|  |  |
| yeast two hybrid constructs: |  |
| AD3894_GAD_SmaI5 | CCA **ccc ggg** TAT GGA CGG CCA GCT CTC CC |
| AD3894_GAD_XhoI3 | CAG **ctc gag** CTA CTG CCC TTT ACG GGC C |
| AD_8008_NdeI5 | **cat atg** GCG ACC AAC AAT ATG C |
| AD_8008_EcoRI3 | **gaa ttc** TTA ACC ACA GTG AGA A |
| AD_ham5_NdeI5 | **cat atg** TCG GTC CCC GGA CAC ATC |
| AD_ham5_BamHI3 | **gga tcc** TTA GAT CAT CTC ACT AT |
| AD_ras2_NdeI5 | **cat atg** GTG GGG AAC AAA CAG G |
| AD_ras2_NdeI3_SLII | **cat atg** TCA TAT AAT CAA Act CTT TC |
| AD_6182_NdeI5 | **cat atg** GCC ATG CTG GCA TC |
| AD_6182_1-136_EcoRI3 | **gaa ttc** CTA CTG GTT CGC ATA G |
| AD_6182_1-360_EcoRI3 | **gaa ttc** CTA TCT TTC GAG TTC T |
| AD_ras1_NdeI5 | **cat atg** GCC AAC AAG TTC ACA AG |
| AD_ras1_EcoRI3_SIMM | **gaa ttc** TCA CAT CAT TAT GCt CTT G |
| SL_0455_GADEcoRI5 | **gaa ttc** ATG AAC TTT GAC TCG GGC ACC |
| SL_0455_GADXhoI3 | **ctc gag** CTA TAT GAT ACC TCC CGG TGG |
| SL_0455_GBKSalI5 | **gtc gac** CCA TGA ACT TTG ACT CGG GCA CC |
| SL_0455_GBKNotI3 | **gcg gcc gc**C TAT ATG ATA CCT CCC GGT GGA T |
| SL_0455_1-200GAD_ EcoRI5 | **gaa ttc** ATG AAC TTT GAC TCG GGC ACC |
| SL_0455_1-200GAD_ XhoI3 | **ctc gag** CAA TGC CGG TAG CAT ATC TTC |
| SL_0455_330-481GAD_ EcoRI5 | **gaa ttc** ATG CCG AGG TCT TAT CGC GG |
| SL_0455_330-481GAD_ XhoI3 | **ctc gag** CTA TAT GAT ACC TCC CGG TGG |
| SL_0455_1-200GBK_ SalI5 | **gtc gac** CCA TGA ACT TTG ACT CGG GCA CC |
| SL_0455_1-200GBK_ NotI3 | **gcg gcc gc**C AAT GCC GGT AGC ATA TCT TC |
| SL_0455_330-481GBK_ SalI5 | **gtc gac** CCA TGC CGA GGT CTT ATC GCG G |
| SL_0455_330-481GBK_ NotI3 | **gcg gcc gc**C TAT ATG ATA CCT CCC GGT GGA T |
